# Feasibility and Acceptability of “Cuida tu Ánimo” (Take Care of Your Mood): An Internet-Based Program for Prevention and Early Intervention of Adolescent Depression in Chile and Colombia

**DOI:** 10.3390/ijerph18189628

**Published:** 2021-09-13

**Authors:** Vania Martínez, Daniel Espinosa-Duque, Álvaro Jiménez-Molina, Graciela Rojas, Paul A. Vöhringer, Mauricio Fernández-Arcila, Carolina Luttges, Matías Irarrázaval, Stephanie Bauer, Markus Moessner

**Affiliations:** 1 Centro de Medicina Reproductiva y Desarrollo Integral del Adolescente (CEMERA), Facultad de Medicina, Universidad de Chile, Santiago 8380455, Chile; alvarojimol@gmail.com (Á.J.-M.); cluttges@med.uchile.cl (C.L.); 2 Millennium Nucleus to Improve the Mental Health of Adolescents and Youths (Imhay), Santiago 8380455, Chile; graciela.rojas.castillo@gmail.com; 3 Millennium Institute for Research in Depression and Personality (MIDAP), Santiago 8380455, Chile; hespinosa@ces.edu.co (D.E.-D.); pvohringer@gmail.com (P.A.V.); mirarrazavald@uchile.cl (M.I.); 4 Facultad de Psicología, Universidad CES, Medellín 050010, Colombia; 5 Millennium Nucleus in Social Development (DESOC), Santiago 8380455, Chile; 6 Facultad de Psicología, Universidad Diego Portales, Santiago 8380455, Chile; 7 Departamento de Psiquiatría y Salud Mental, Hospital Clínico Universidad de Chile, Santiago 8380455, Chile; 8 Mood Disorders Program, Tufts Medical Center, Tufts University, Boston, MA 02155, USA; 9 Departamento de Psicoanálisis, Universidad de Antioquia, Medellín 050010, Colombia; mauricio.fernandez@udea.edu.co; 10 Center for Psychotherapy Research, University Hospital Heidelberg, 69120 Heidelberg, Germany; stephanie.bauer@med.uni-heidelberg.de (S.B.); markus.moessner@med.uni-heidelberg.de (M.M.)

**Keywords:** adolescent, depression, prevention, internet-based interventions, e-mental health

## Abstract

The rapid internet penetration in Latin American countries has made it possible to implement digital mental health interventions. “Cuida tu Ánimo” (Take Care of Your Mood) is an internet-based program for the prevention and early intervention of depression in adolescents. A pilot study was conducted in Chile and Colombia to study the feasibility and acceptability of the program and estimate its effects. There were 199 participants (53.3% women; mean age = 14.8 years, *SD* = 1.0) recruited from two schools in Chile and two schools in Colombia. Qualitative and quantitative methods were applied for data collection and analyses. Although the levels of acceptance were moderate to high across all variables, adherence was lower than expected. The participants deemed important for an intervention of this type offered a higher level of interaction with team members through internet-based and face-to-face activities. Post-intervention outcomes show a reduction in depressive and anxious symptoms in adolescents in Chile, while there were no significant changes in the level of symptomatology in adolescents in Colombia. The women used the program more than the men. Results show the need to improve the intervention by increasing its levels of customization and developing strategies to achieve better adherence. The contradictory results of the program in Chile and Colombia suggest the importance of other variables beyond the content of the intervention, such as the setting or context of the intervention.

## 1. Introduction

Depression in adolescents is a worldwide challenge that entails huge individual and social costs [1]. Depression and sub-syndromal depressive symptoms in adolescents are associated with persistent functional impairment, physical health problems, and other psychiatric disorders, learning difficulties, risky behaviors, substance misuse, and difficulties in interpersonal relationships [2,3,4]. The severity of depressive symptoms is also associated with increased suicidal ideation and behavior [5,6] as well as poorer quality of life and poorer mental health in adulthood [7].

Epidemiological studies show that depression is common among adolescents, with up to a 25% lifetime prevalence before adulthood [8]. In an epidemiological study conducted in Santiago (Chile), the annual prevalence of depression associated with social disability in adolescents between 12 and 18 years was 7.8% [9]. In Colombia, the lifetime prevalence of any depressive disorder in adolescents was 2.4% [10]. In Medellín (Colombia), a study using the DSM-IV criteria showed that the prevalence of depression reached 13.1% in adolescents [11].

Despite the existence of effective treatments for depression, there is evidence for low rates of help seeking among adolescents [12]. When assistance is given, proper diagnosis and/or treatment are not always provided [12]. In this context, it is important to develop effective strategies for prevention and early intervention.

Interventions aimed at reducing the burden of depression have become a priority public health need. These interventions face great complexity due to the multiple variables associated with depressive symptoms, the high costs of treatment, and the specific characteristics of this problem in adolescence [13]. This stresses the importance of preventive interventions and early interventions for depression [1]. Early intervention for depression aims at influencing the early stages of the morbid process, given that early depressive symptomatology is a stronger predictor of depressive disorders than other risk factors [14].

Growing evidence suggests that targeted prevention and early intervention programs in adolescent depression produce larger effect sizes than universal programs in improving psychological wellbeing or preventing mental disorders [15]. Studies on depression prevention in school settings show small effect sizes (*g* = 0.23 immediately after the intervention and *g* = 0.11 at 12-month follow-up). In addition, universal programs have worse outcomes than indicated prevention programs [16].

In Santiago (Chile), a universal preventive intervention investigated in a sample of 2512 secondary students from 22 schools failed to demonstrate its efficacy in reducing depressive symptoms [17]. The authors of this study argued that school programs to counteract adolescent depression might fail when delivered in whole classrooms because they target an entire class instead of a specific population. Likewise, these programs can be unattractive to participants and do not reach the intensity required to achieve significant effects on depression outcomes.

In recent years, numerous internet-based interventions have been developed worldwide and have proven to be useful and effective [18,19,20,21]. Several studies have shown that these types of interventions are effective in reducing depressive symptoms [22]. While there is a growing interest in internet-based interventions for mental disorders in developing countries, very few studies have been conducted on this topic [23], especially in Latin America [24].

In Latin American countries, such as Colombia and Chile, developing and testing these types of interventions is critical given the high prevalence of depression [10,25]. In addition, it is expected that the general population, including adolescents, will be able to access this type of intervention, as the levels of internet connectivity have been increasing exponentially in recent years [26,27].

More evidence should be generated on interventions designed specifically for adolescents to minimize the impact of risk factors and promote healthy lifestyles at this stage of development. The internet offers the opportunity to provide this type of intervention given its high penetration in the daily lives of adolescents [18,28]. The implementation of internet-based prevention and early intervention programs represents a strategic opportunity to reduce gaps in mental healthcare for people in developing countries in a cost-effective way [23,29,30]. While internet-based preventive programs for depression have mainly been studied in adult rather than in adolescent populations, preliminary evidence suggests they could be a helpful alternative [19,20,21,31,32,33,34,35].

Internet-based interventions developed for a school setting could provide greater flexibility, as there is no need to be physically present during the intervention, and it is easy to provide tailored support according to individual needs. However, a major limitation of digital programs is the high dropout rate [28,36]. A systematic review of research addressing adherence to internet-based interventions revealed an average dropout rate of 50% [37]. Thus, the inclusion of adolescents’ preferences and perspectives in the design and development of internet-based programs is crucial to provide acceptable and effective interventions [38].

The objective of this study was to evaluate the feasibility and acceptability and estimate the effects of an internet-based program for the prevention and early intervention of adolescent depression in high school students implemented in Santiago (Chile) and Medellín (Colombia).

## 2. Materials and Methods

A pilot study was conducted with adolescents in Chile and Colombia to study the feasibility and acceptability of the “Cuida tu Ánimo” (CTA) (Take Care of Your Mood) program and estimate its effects. A mixed methodology (qualitative and quantitative) was applied for data collection and analysis.

### 2.1. Sample and Setting

Four schools (two schools in each country) were invited to participate. In Chile, the choice of schools was for convenience since there was previous contact with them. In Colombia, the schools were suggested by the Municipal Secretariat for Education. In Chile, they were subsidized private schools, and in Colombia, they were public schools. Only schools with a school psychologist or school counselor were eligible to participate, and the school’s principal had to consent to participation. Before inclusion in the study, participants had to provide written informed consent. For students younger than 18 years old, informed consent from their parents or primary caregivers was required. In the schools, a face-to-face induction to the program was provided to introduce CTA and provide information about the usage and access to the intervention. In order to use the program, participants had to register on the web platform.

A total of 517 adolescents (271 from Chile and 246 from Colombia) in grades 8 to 11 in Chile and 6 to 10 in Colombia were invited to participate. The final sample consisted of 199 participants (106 women and 93 men; 146 (73.4%) from Chile and 53 (26.6%) from Colombia).

### 2.2. Intervention

CTA is an internet-based program for the prevention and early intervention of adolescent depression (www.cuidatuanimo.org, accessed on 7 September 2021). The program was developed based on the experience and results of previous studies by the research team, which were conducted in another setting [39], sampled another population [40], or targeted a different mental health problem [41].

The students were registered in the program in their school’s computer lab under the supervision of the research team staff. Participants were able to access the program from computers or smartphones without any time restrictions. To promote use, several visits were made to schools that participated in the study, in which research staff members involved in the program distributed bracelets and handouts with information about the program along with other merchandising products. The participants were able to use the program for six months.

The program followed an individualized approach in which the intensity of support was matched to the participants’ actual needs (i.e., depressive symptomatology).

The CTA program included the following components:

1. Psycho-educational information (Learn): The program offered information on depression (what it is, symptoms, and causes), healthy lifestyle habits, emotion regulation, social support networks, and cognitive-behavioral techniques (behavioral activation, problem-solving, and cognitive restructuring).
2. Mood monitoring and feedback (How did you feel?): This component included (a) mood monitoring: every two weeks, participants received an e-mail with a link to an online questionnaire (items 1, 2, and 9 of the Patient Health Questionnaire-9 (PHQ-9)) [42]; (b) individualized feedback: every time the adolescents answered the mood monitoring, the program automatically sent a feedback message based on a predefined algorithm according to the level of reported depressive symptoms (minimum, mild, moderate); and (c) alarms: if adolescents reported thoughts that they would be better off dead or hurting themselves (item 9 of PHQ-9 >1) [42], the program team received an alert and contacted the adolescents through the school coordinator to offer them the possibility of scheduling an individual chat or phone session with a therapist. In that session, the need to inform parents or primary caregivers and the need for a face-to-face intervention were assessed.
3. Forums: The program offered a space for peer interaction moderated by a mental health professional on general mental health topics (e.g., mood-boosting songs).
4. Individual chat or phone call: An individualized support space was offered, accompanied by a program professional. Teenagers were able to book a 45-min appointment with a psychologist. Adolescents were able to access this component at any time during the program, and when they reported moderate or severe symptoms during monitoring, they were encouraged to request this component in the feedback message.
5. News blog: There was a regularly updated blog with news and general information on mental health and related topics. Participants were able to comment on and discuss the contents.
6. Emergency: This section provided adolescents with a crisis plan and general information for dealing with acute crises (e.g., helplines, support networks, crisis management activities).
7. Contact: Teenagers were able to contact program professionals at any time via e-mail.

### 2.3. Measures

#### 2.3.1. Primary Outcome

The primary outcome measure was depressive symptomatology, measured with the PHQ-9 [42]. The PHQ-9 is a 9-item, self-report questionnaire used for the evaluation of depressive symptoms in adolescents according to criteria set out in the Diagnostic and Statistical Manual of Mental Disorders (DSM). This questionnaire is composed of a 4-point Likert scale (from 0 = not at all to 3 = nearly every day). Higher scores indicate more severe depressive symptoms. This outcome measure was assessed at baseline and after 6 months.

#### 2.3.2. Secondary Outcomes

In addition, anxious symptoms were assessed as a secondary outcome given their strong association and comorbidity with depressive symptomatology [43]. Anxious symptoms were measured with the Generalized Anxiety Disorder-7 questionnaire (GAD-7; [44]). This is a 7-item, self-report questionnaire to assess the presence and magnitude of generalized anxiety symptoms according to DSM criteria. It is composed of a 4-point Likert scale ranging from 0 (not at all) to 3 (nearly every day). Higher scores indicate more severe anxiety symptoms.

Health-related quality of life (HRQoL) was measured with the KIDSCREEN-27 questionnaire [45], which includes five dimensions and the 10-item general index score. Higher scores indicate better HRQoL.

The Self-Stigma of Depression Scale (SSDS) [46] was used to assess the extent to which a person holds stigmatizing attitudes towards themselves due to having depression. It consists of 16 items. Responses are measured on a 5-point Likert scale (ranging from one (strongly agree) to five (strongly disagree)). Items are coded so that a higher score indicates greater self-stigma.

Assessments for all measures took place at baseline and after 6 months.

#### 2.3.3. Program Utilization

The usability of the intervention was evaluated considering the number of clicks on the page and the use of modules.

Adherence to the monitoring was defined by the research team as a minimum of one-third of completed monitoring assessments (five responses).

#### 2.3.4. Acceptance of the Program

Students who actively used the program were surveyed about their acceptance of the program and their satisfaction with the support that CTA offered to them. Acceptance was evaluated through 7 questions with a 4-point Likert scale ranging from 1 (strongly disagree) to 4 (strongly agree). Higher scores indicate more satisfaction with the program. Good satisfaction with the program was defined as a score of 3 or 4. A question on overall satisfaction with the program was asked on a scale of 1 to 10. A higher score indicates more satisfaction with the program. In addition, the students answered two open-ended questions about positive aspects of the program and recommendations for improvement.

Finally, four focus groups were held in Chile (*n* = 2) and Colombia (*n* = 2) to explore the adolescents’ expectations, their user experience, and their evaluation of the program’s content and accessibility as well as to obtain suggestions to improve the program.

### 2.4. Data Analysis

Descriptive analyses of socio-demographic and baseline outcome variables were carried out. In addition, differences in outcome variables between the baseline and six-month assessment were calculated using *t*-test with two tails, and uneven variance was applied, with a *p*-value set at 5%. Program-use rates and program-acceptability percentages were calculated. As a dependent variable, we stratified the sample into “high webpage users” (students placed in the 75% percentile or above of webpage usage). Then, a multivariate logistic regression model was developed using this binary variable as the main outcome. Finally, a descriptive content analysis was conducted of the central themes reported by the adolescents in the open-ended questions on positive aspects of the program and recommendations for improvement as well as in the focus groups.

### 2.5. Ethical Considerations

All procedures were approved by the Ethics Review Boards of both Universities participating in this study. Ethical approval was obtained from the Ethics Committee of Human Research of the Faculty of Medicine of the Universidad de Chile (Chile) and the Institutional Ethics Committee of Human Research of the Universidad CES (Colombia).

Adolescents with severe depressive symptoms and/or suicide risk at the baseline assessment were referred to face-to-face evaluation with their parents or primary caregivers in coordination with a school psychologist to determine the need for referral to face-to-face treatment.

## 3. Results

A total of 517 adolescents were invited to participate. A different recruitment rate was observed depending on the country. Of the adolescents invited, 52.3% accepted in Chile and 21.5% in Colombia. The final sample of this pilot study included 199 participants (106 women and 93 men; mean age = 14.8 years, *SD* = 1.0, range: 13–18; mean years of schooling = 9.1, *SD* = 0.8) recruited from four schools in Colombia and Chile. Table 1 shows the characteristics of participants in the CTA program at baseline in each country.

The PHQ-9 score at baseline was 8.9 (*SD* = 5.7) and 7.2 (*SD* = 4.8) at the post-intervention assessment after six months (Table 2). The PHQ-9 score shows a significant decrease in depressive symptoms post-intervention due to the change in the Chilean sample. There was a significant difference between Chile and Colombia in the depressive symptoms at six-month follow-up (mean difference 1.4, *SD* = 2.07, *p* < 0.000).

Table 3 shows the results of the participants in secondary outcome scales at baseline and at six months in each country. There was an increase in the levels of Self-Stigma of Depression in the Colombian sample and a decrease in anxious symptomatology post-intervention due to the change in the Chilean sample.

### 3.1. Program Utilization

Participants received a total of 16 e-mails each asking them to complete the mood monitoring. Participants answered on average 1.8 (*SD* = 2.9) monitoring assessments. Almost half of the participants (48%, *n* = 96) did not respond to any monitoring assessments, 17% (*n* = 34) responded to only one, and 7% (*n* = 13) responded to seven or more monitoring assessments. Only 15% of the participants (*n* = 29) made an expected use of the program, i.e., they responded to at least one-third of the monitoring assessments (five responses).

Concerning the use of the CTA website, participants clicked on the platform six times on average (*SD* = 9.4), with a maximum of 55 clicks. This level of use was similar in both countries. The sex of the participant was the principal predictor of high website usage (i.e., those participants who are in the 75th percentile of highest use). Being a woman increased the probability of being placed in the 75% percentile or above of webpage usage by almost three times (*OR =* 2.9). Impairment—at either baseline or six months—was not associated with the intensity of website usage.

In total, 102 alarm notifications were sent to the CTA team following the monitoring assessments. Forty-three participants (29 in Chile, equivalent to 19.9% of this group, and 14 in Colombia, equivalent to 26.4% of this group) activated the alarm at least once, indicating the possibility of suicidal thoughts throughout the intervention. These participants were contacted through the school coordinator to offer them the possibility of scheduling an individual chat or phone session with a therapist. However, only five (one in Colombia, four in Chile) utilized that counseling appointment. One adolescent in Chile was referred to face-to-face care with his parents.

### 3.2. Acceptability of the Program

A total of 103 participants (51.8%) answered the acceptability and satisfaction questionnaire. The overall rating of the program was 7.1 (*SD =* 2.1) (score range: 1–10). Most participants were satisfied with the program (82.5%) and would recommend it to another adolescent (77.7%). A total of 60.8% of the participants considered the program useful, and 67.6% stated that it met their expectations. Regarding technical aspects and their usage of the website, 69.9% of the participants rated it as entertaining, while 86.1% stated that it was easy to use and comfortable. Likewise, 62.1% of the participants believed that they learned new things.

In the open-ended questions, the adolescents indicated that they believed the program was a place for emotional help and expression and that their participation allowed them to learn about depression. None of them stated that participation was detrimental. However, they recommended more interaction with team members through online and face-to-face activities.

### 3.3. Qualitative Evaluation of the Program

In the focus groups held in Colombia and Chile, the participants highlighted some positive aspects of the program.

**Source of support.** Participants noted that the CTA program was valuable because they regarded it as a source of support. They greatly appreciated that the program was guided by specialists who cared about them and were available to support them: “[CTA] gives us the chance to talk to professionals”. The availability of support and contact with mental health professionals, if required, along with “concern about young people’s mental health who are more likely to suffer” were among the main factors related to participants’ satisfaction. In this regard, one user also noted: “I appreciate the program’s intention to help, e-mails arrived frequently and help is offered through scheduling a chat appointment”.

**Useful information.** The participants also stated that the CTA website provided useful information on depression and was a useful tool for depression detection: “It’s a good option for people who are depressed and try to overcome their state”; “This program has given me a broad knowledge of depression, what you can do to prevent it and how to help people”.

**Entertaining and informative.** Participants also stated that they found the psycho-educational videos on the website entertaining and informative.

**Promotes awareness of how they feel.** They valued that the monitoring assessment allowed them to become aware of how they were feeling. In this regard, the participants said that the program informed insights into and reflection on their mood: “It was interesting to realize how I was with the survey results and the fact that I think very little about that”. This may not only help them to express negative emotional states but also allows them to handle negative emotions.

On the other hand, participants made several suggestions to improve the CTA program.

**Design of the website.** They underlined aspects associated with the design of the website, its attractiveness, accessibility, content, and style of interaction: “I think the website should be more appealing, interactive, and entertaining, with less text”.

**Preference for smartphone access.** Most participants stated that they preferred accessing the site on smartphones instead of computers, although they recognized that sometimes they have difficulties accessing the internet.

**Use of e-mails.** Participants mentioned that e-mails are not an appropriate way of contacting them, and that contact “should be more direct”. In this regard, they recommend complementing the program with other sites and social media platforms, such as Facebook and Instagram. “Normally, we don’t check our e-mails that much, we are more likely to log on social networks, not e-mail”.

**Diversifying the questions.** With respect to the monitoring assessments, the participants recommended diversifying the questions and suggested that the program “should ask us more questions about what we do outside of school, or about what we do in our spare time”.

**Response to alarms.** The participants also highlighted some recommendations regarding the response of the professional staff to the system alarm. According to participants, the staff should be available to give an individual and “timely solution” to the specific needs of each user, “to act immediately, because that’s when things happen”. In this regard, the staff should be more active in contacting adolescents, which would require implementing a quick contact option for those who report difficulties: “Not asking the students to make an appointment, but instead to schedule it themselves”; “It would be more useful if, while we’re answering [the survey], and you saw we are not doing fine, you directly contacted us”.

**True stories.** When asked specifically about what the program should include, the main recommendations were to show true stories of adolescents on the website and incorporate “videos about the experience of other people”.

**Personal expression and group interaction.** The participants also suggested incorporating more spaces for communication and opportunities for personal expression and group interaction, and “to have a place where you can write what you feel”; “I would write something like… a situation, I don’t know, last week my parents yelled at me, I felt bad, now I feel fine, so it’s like… more [expression options]”; “A discussion forum could be useful to show different realities, that not everybody has. I mean, maybe everyone has the same issue but not everyone experiences them in the same way, so that would help”.

**Games and videos.** The participants also suggested introducing games and videos to improve the program’s level of interactivity and entertainment value: “I would like it to be more fun, with games and videos”.

**Face-to face activities.** Another important issue for adolescents is closeness and face-to-face interaction. Some participants expected to have more availability of face-to-face activities.

**Dissemination.** An important dimension underlined by the participants about the scope of the CTA program is its dissemination among the target population. The program should not only have greater promotion within the participants’ schools but also on social media and other platforms.

## 4. Discussion

The results yielded by this pilot study showed a change in depressive and anxious symptoms in Chilean adolescents, while there were no significant changes in the level of symptomatology of Colombian adolescents. This could be explained by contextual reasons. The participating schools in Colombia had a more vulnerable population than the Chilean schools. They were exposed to greater psychosocial risks and historically affected by multiple types of violence. In addition, institutional constraints in Colombia, such as frequent strikes and few extracurricular opportunities, affected the intensity of some of the proposed activities.

On the other hand, measures of HRQoL stayed at the same average value after the intervention. In addition, none of the outcome measures allowed us to predict levels of program use. This calls into question the hypothesis that adolescents with more severe symptomatology make greater use of programs of this type.

Two specific elements in the Colombian sample were also found: the low rate of agreement to participate in the program and the increased levels of self-stigma of depression after the program. Although in Colombia, it is necessary to continue studying the stigma related to mental disorders and its associated elements [47], some research considers stigma as a cultural barrier in mental health [48], revealing high levels of stigma in adolescents in Medellín, compared to those in Santiago, Chile [49]. In addition, in the process of implementing the program, stigmatizing ideas were identified from some of the adolescents who did not agree to participate in the study towards those who did agree to participate. These aspects may be related to the low interest in participating and the increase in negative ideas related to depression.

Regarding the feasibility of use and acceptability of the program, it was found that the program was used and accepted by the adolescents. Although the levels of satisfaction were moderate to high in all measured items (62.1–82.5%), utilization rates were low overall. These results are in line with studies that challenge the assumption that adolescents prefer internet-based interventions [50]. Correspondingly, we found a relatively low adherence to the program, a common problem of internet-based interventions in mental health [28,36,37].

A study on the feasibility and acceptability of a monitoring program for patients in treatment for depression conducted in Chile showed similar results: participants showed a high level of acceptance of the program and considered it a source of support and benefit but used it substantially less than expected [40]. This is not unusual given that prevention programs tend to have lower adherence than treatment, as participants do not experience as much suffering [37]. Nevertheless, it is important to note that adolescents tend to value the program, do not consider it harmful, and find it useful to be part of it and receive information related to depression.

The present study also shows that female adolescents made greater use of the program. The sex of the participants was the main predictor of website use. This result is consistent with research showing a gender difference in the use of digital interventions, with women being more receptive to behavior change strategies [51].

In addition to the factors mentioned above, the opportunity to have individual chat or phone sessions was considered useful even if the participants did not use this resource much. In this regard, perceived staff interest and concern for users emerges as one of the main factors associated with satisfaction. Several studies have argued that regular contact may give people a feeling of “social connection” [52]. In other words, the feeling that there is someone available who can be accessed in difficult situations seems to act as a factor of psychological support [40].

Concerning suggestions for improving the program, the adolescents consider it essential to improve the attractiveness and design of the website. In this regard, the key aspects of the “Persuasive Systems Design Model” (PSDM) could be incorporated into the CTA program to improve adolescent use, adherence, and satisfaction [38]. Many of the participants’ suggestions coincide with the principles of the PSDM [40]: to promote interactions between users, to include a group communication platform, and to incorporate more personalized content.

In addition to improving the attractiveness and persuasiveness of the content of the website, the adolescents consider it necessary for them to be involved in the design of the intervention to encourage participation. The results of the focus groups pose the challenge of incorporating internet-based program activities that reflect greater proximity to adolescents and are more playful in nature. The adolescents also consider it important to have a higher level of presence and interaction with team members and other peers through the internet, engage in more face-to-face activities, and receive relatable information and stories. This type of strategy could offer adolescents a space of greater trust and closeness, as has been reported in other studies [50].

With regard to technical recommendations, participants mentioned that e-mails are not an adequate way to contact them. This requires developers to use contact methods that are more familiar to young people and to prepare programs for migration to new types of networks and technologies that are being introduced while maintaining information security and privacy. Given the rapid penetration of smartphones in Latin America and elsewhere, the integration of app-based interventions may offer the potential to provide more widely distributed mental health care [53].

In order to test the efficacy of the intervention and investigate the specific subgroups of adolescents who are most likely to use and benefit from the CTA program, a randomized controlled trial (RCT) needs to be conducted.

Overall, this pilot study showed moderate to high levels of acceptance; however, adherence was lower than expected. This suggests that it is necessary to not only improve the intervention but also to increase its level of personalization so that students have an experience of greater identification and closeness so that users can take ownership of it and benefit to a greater extent.

The program could be implemented in a variety of contexts, which opens up the possibility of thinking about interventions with a broader scope. This type of experience makes it possible to evaluate the feasibility of overcoming cultural differences and implementing interventions at the Latin American level, taking advantage of the shared language. However, the contradictory results of the program in Chile and Colombia suggest the importance of other variables beyond the content of the intervention, such as the setting or context of the intervention.

In conditions of greater psychosocial adversity, it is recommended that: (a) the professionals implementing the intervention work directly in the institution to increase the familiarity and intensity of the process, (b) articulate the intervention with other institutional projects and activities, and (c) increase awareness campaigns on mental health and stigma.

## 5. Conclusions

This pilot study conducted in two schools in Santiago, Chile, and two schools in Medellín, Colombia, showed high levels of acceptance of an internet-based program—“Cuida tu Ánimo” (CTA) (Take Care of Your Mood)—for the prevention and early intervention of depression in adolescence. However, our findings showed the need to improve the intervention by increasing its levels of customization, developing strategies to achieve better adherence, and considering the setting or context of the intervention. Furthermore, this feasibility study should be followed by an RCT to study the efficacy of CTA.

## Figures and Tables

**Table 1:** Characteristics of participants at baseline in each country.

	Colombia	Chile	Total
Participants, *n* (%) by school, *n*	*n* = 53 (26.6) School A: 26 School B: 27	*n* = 146 (73.4) School C: 75 School D: 71	*n* = 199
Women, *n* (%)	32 (60.4)	74 (50.7)	106 (53.3)
Age in years mean (*SD*)	14.9 (1.1)	14.7 (1.0)	14.8 (1.0)
Years of schooling mean (*SD*)	8.9 (0.8)	9.1 (0.7)	9.1 (0.8)

*SD*, standard deviation.

**Table 2:** Mean PHQ-9 score at baseline and 6-month follow-up and mean differences by country.

	PHQ-9 Score			
	Baseline Mean (*SD*), *n*	6 Months Mean (*SD*), *n*	Mean Difference (*SD*)	*p*-Value
**Colombia**	8.7 (5.0), 53	8.2 (5.2), 44	0.5 (1.0)	0.650
**Chile**	8.9 (6.0), 146,	6.7 (4.7), 107	2.2 (0.7)	0.001
**Total**	8.9 (5.7), 199	7.2 (4.8), 151	1.7 (1.6)	0.003

**Table 3:** Secondary outcomes at baseline and at 6 months.

	Colombia		Chile		Total	
	Baseline Mean (*SD*) *n* = 53	6 Months Mean (*SD*) *n* = 44	Baseline Mean (*SD*) *n* = 146	6 Months Mean (*SD*) *n* = 107	Baseline Mean (*SD*) *n* = 160	6 Months Mean (*SD*) *n* = 151
GAD-7	5.7 (4.4)	6.1 (4.2)	7.1 (4.7) **	5.2 (3.9) **	6.7 (4.7) *	5.5 (4.0) *
SSDS	54.2 (5.9) ***	63.1 (5.8) ***	49.0 (12.0)	47.4 (11.4)	50.4 (11.0)	52.0 (12.4)
Physical wellbeing ^a^	41.5 (11.3)	42.5 (10.8)	43.3 (11.8)	43.0 (12.0)	42.8 (11.7)	42.9 (11.7)
Psychological wellbeing ^a^	44.6 (10.9)	44.2 (10.8)	44.5 (11.5)	46.9 (11.1)	44.5 (11.3)	46.1 (11.0)
Autonomy and parent relation ^a^	46.1 (7.2)	45.2 (9.3)	48.0 (10.9)	49.1 (11.0)	47.5 (10.1)	48.0 (10.7)
Peers and social support ^a^	43.4 (10.5)	44.4 (9.0)	51.9 (11.2)	52.2 (10.8)	49.6 (11.6)	49.9 (10.9)
School environment ^a^	48.6 (10.6)	47.6 (9.5)	47.3 (8.4)	47.7 (8.1)	47.7 (9.0)	47.6 (8.5)
Health-related quality of life index ^b^	41.6 (5.1)	40.8 (4.9)	41.6 (4.5)	41.2 (6.2)	41.6 (4.7)	41.1 (5.8)

* *p* = 0.0165, ** *p* = 0.005, *** *p* = 0.001. ^a^ KIDSCREEN-27, dimension score. ^b^ Uses 10 items derived from the 27-item version of KIDSCREEN.

## Data Availability

The data presented in this study are available on request from the corresponding author.
